# Endoplasmic Reticulum Stress Underlies Nanosilver-Induced Neurotoxicity in Immature Rat Brain

**DOI:** 10.3390/ijms232113013

**Published:** 2022-10-27

**Authors:** Beata Dąbrowska-Bouta, Grzegorz Sulkowski, Magdalena Gewartowska, Lidia Strużyńska

**Affiliations:** 1 Laboratory of Pathoneurochemistry, Department of Neurochemistry, Mossakowski Medical Research Institute, Polish Academy of Sciences, 5 Pawińskiego Str., 02-106 Warsaw, Poland; 2 Electron Microscopy Research Unit, Mossakowski Medical Research Institute, Polish Academy of Sciences, 5 Pawińskiego Str., 02-106 Warsaw, Poland

**Keywords:** AgNPs, ER stress, developmental neurotoxicity, unfolded protein response, UPR pathway

## Abstract

The growing production of silver nanoparticles (AgNPs), and their widespread use in medical and consumer products, poses a potential threat to the environment and raises questions about biosafety. Immature organisms are particularly susceptible to various insults during development. The biological characteristics of immature organisms are different from those of adults, and dictate the consequences of exposure to various toxic substances, including AgNPs. Nanoparticles are highly reactive and can easily cross the blood–brain barrier (BBB) to accumulate in brain tissues. It is therefore important to investigate the molecular mechanisms of AgNP-induced neurotoxicity in the developing brain. Immature 2-week-old rats were exposed to a low dose of AgNPs (0.2 mg/kg b.w.) over a long period. Subsequently, brain tissues of the animals were subjected to ultrastructural and molecular analyses to determine endoplasmic reticulum (ER) stress. Ultrastructural markers of ER stress, such as pathological alterations in the ER and elongated forms of mitochondria accompanied by autophagy structures, were confirmed to be present in AgNP-exposed rat brain. Evidence for induction of ER stress in neurons was also provided by molecular markers. Upregulation of genes related to the ER-stress-induced unfolded protein response (UPR) pathway, such as GRP78, PERK, and CHOP ATF-6, was observed at the transcriptional and translational levels. The results show that prolonged exposure of immature rats to a low dose of AgNPs during the developmental period leads to induction of ER stress in the neurons of the developing brain. Simultaneously, in response to AgNP-induced ER stress, neurons promote protective mechanisms that partially compensate for ER stress by regulating the biodynamic processes of mitochondria and autophagy.

## 1. Introduction

Extensive production and use of many different types of metal nanoparticles (NPs), including silver nanoparticles (AgNPs), has occurred over the past few decades. Since AgNPs possess unique physical and chemical properties, such as strong antimicrobial properties, they are frequently used for medical purposes and are incorporated into many consumer products [1]. AgNPs are also extensively included in products dedicated to infants and children [2], which raises questions about health and safety. The general toxicity and neurotoxicity of AgNPs have been already confirmed in an array of studies that revealed alterations in locomotor activity [3] and impairment of spatial cognition [4]. Studies on the molecular mechanisms of AgNP-induced neurotoxicity in adult rat brain have indicated overproduction of reactive oxygen species (ROS), apoptotic/necrotic neuronal cell death (for reviews see: [5,6]) and induction of autophagy [7].

Current knowledge on the developmental neurotoxicity of AgNPs has been recently collected and discussed [8]. However, the dose–response and the mechanisms of neurotoxicity in young organisms are still poorly defined, particularly after prolonged exposure. Previous studies have confirmed persistent accumulation of silver in brain tissue of AgNP-exposed immature rats [9], in which decreased density of glutamate N-methyl-D-aspartate (NMDA) receptors, together with decreased expression of several NMDAR complex-related proteins, was identified [10]. Furthermore, downregulation of the GluN2B-PSD95-nNOS-cGMP signaling pathway has been confirmed [10]. This pathway maintains LTP/LTD processes underlying learning and memory formation during development. These results demonstrate that AgNPs exert toxic effects in neurons, particularly glutamatergic neurons. Moreover, our previous studies on the immature AgNP-exposed rat brains have revealed the existence of ultrastructural alterations in endoplasmic reticulum (ER) and concomitant downregulation of neuronal glutamate transporter EAAC1, which may be related to impaired protein folding in the ER compartment [11].

The endoplasmic reticulum (ER) is organized into subcompartments constructed from a dynamic network of tubules and cisternae. This organelle is involved in cellular processes such as protein and lipid synthesis, gluconeogenesis, calcium storage and release, as well as the generation of autophagosomes and peroxisomes [12]. The molecular chaperones in the ER prevent aggregation of the proteins, playing an important role in their folding and maturation. ER homeostasis provides proper protein formation of three-dimensional structures through translational and posttranslational modifications, and a balance between the ER protein load and decomposition [13]. The ER can be disturbed by many factors such as high protein demand, inflammatory cytokines, or environmental toxins, leading to ER stress [14]. To counteract ER stress, there is a cellular mechanism of adaptive response known as the unfolded protein response (UPR), which is initiated by three ER transmembrane proteins: inositol requiring 1 (IRE1), PKR-like ER kinase (PERK), and activating transcription factor 6 (ATF6). Under normal conditions, these regulatory proteins are kept inactive by the chaperone protein GRP78 (glucose-regulated protein 78 kDa, also known as BiP). Under stressor conditions, GRP78 dissociates and activates regulatory proteins and their downstream pathways in order to reestablish ER homeostasis via distinct functions such as the adaptive response, feedback control of the ER, and cell fate regulation [14,15]. The purpose of the UPR-mediated adaptive response is: (i) to upregulate protein folding and handling efficiency, (ii) to suppress translational processes in order to minimize further overload of the ER, (iii) to increase ER-associated protein degradation and the autophagy process, thereby promoting clearance of damaged proteins, and (iv) to induce protective mitochondrial elongation through mechanisms such as stress-induced mitochondrial hyperfusion (SIMH) [16]. After recovery of ER homeostasis, negative regulation of UPR is activated. When the UPR can relieve the ER stress, the cell will survive. In situations in which the UPR is unable to reduce ER stress and restore cell homeostasis, it promotes cell death. The fate of cells exposed to ER stress is regulated by the UPR via both apoptotic and anti-apoptotic pathways, playing an important role in the pathogenesis of ER-stress-related disorders [15,17].

Although the neurotoxicity of AgNPs has been extensively studied in adult animals, it has not been significantly investigated in immature organisms. Therefore, the present study was designed to determine whether a low dose of AgNPs (0.2 mg/kg b.w.) administered continuously for 3 weeks to immature rats will induce cellular stress in the brain. Ultrastructural analyses of brains, as well as molecular analyses of selected ER stress markers such as GRP-78, PERK/phospho-PERK, ATF6, and CHOP, were performed on tissues of exposed animals. Silver citrate (Ag^+^) was used as a positive control to compare the results vs. particulate form of silver.

## 2. Results

### 2.1. Silver Concentrations in Brain of Exposed Rats

Exposure to a low dose of either AgNPs or silver citrate did not influence the body weight or the general appearance of the animals. Inductively coupled plasma mass spectrometry (ICP-MS) analyses of brain samples of control rats did not reveal silver concentrations above the detection limit (0.011 mg/kg wet weight). In brain samples obtained from exposed rats, silver concentrations were 0.15 ± 0.01 mg/kg and 0.23 ± 0.03 mg/kg for AgNP-treated and Ag citrate-treated (Ag^+^-treated) rats, respectively.

### 2.2. Ultrastructural Characteristics in Neurons Indicative of ER Stress

The first objective of the study was to analyze whether AgNPs administered at a low dose, and accumulating in the brain of immature rats after prolonged exposure, induce damage to cellular structures.

**Figure 1:**
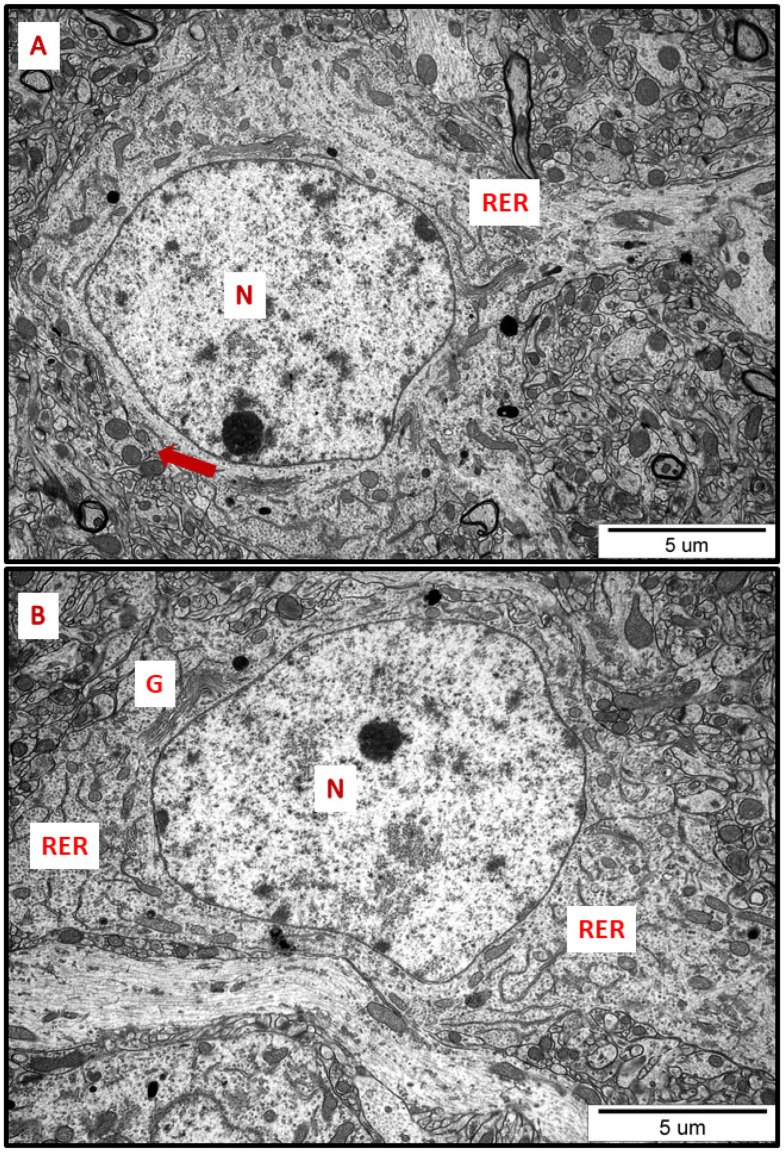
Representative TEM micrographs of cerebral (**A**) and hippocampal (**B**) sections from control animals. Normal ultrastructure of neurons is visible; RER—rough endoplasmic reticulum; N—nucleus; Golgi apparatus (G); mitochondria (arrows). Images are typical for each of the 3 examined animals per group.

**Figure 2:**
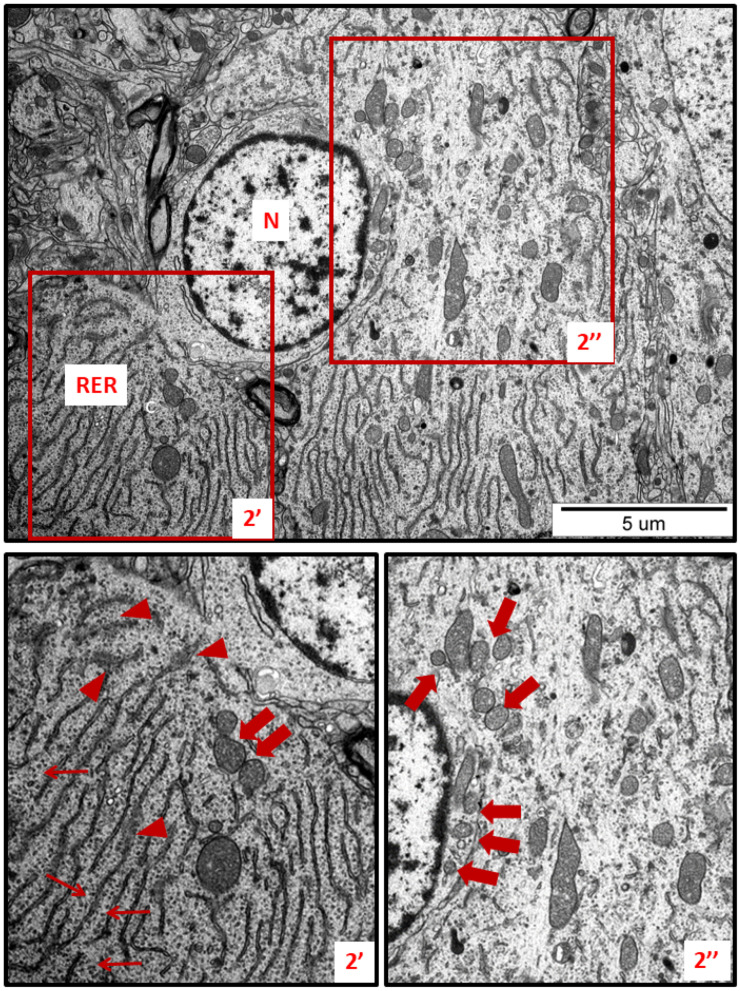
Representative TEM micrographs showing ultrastructural changes in subcellular structures of neurons in cerebral sections obtained from AgNP-treated rats. RER—rough endoplasmic reticulum; N—nucleus. Enlarged and thickened cisternae of RER (2′); swollen fragments of RER with blurred structure (arrowheads); ribosomes detached from RER lying free in the cytoplasm (thin arrows); fragmentation of mitochondria (thick arrows). Insets (2′, 2″) were magnified 2×. Images are representative for each of the 3 animals.

**Figure 3:**
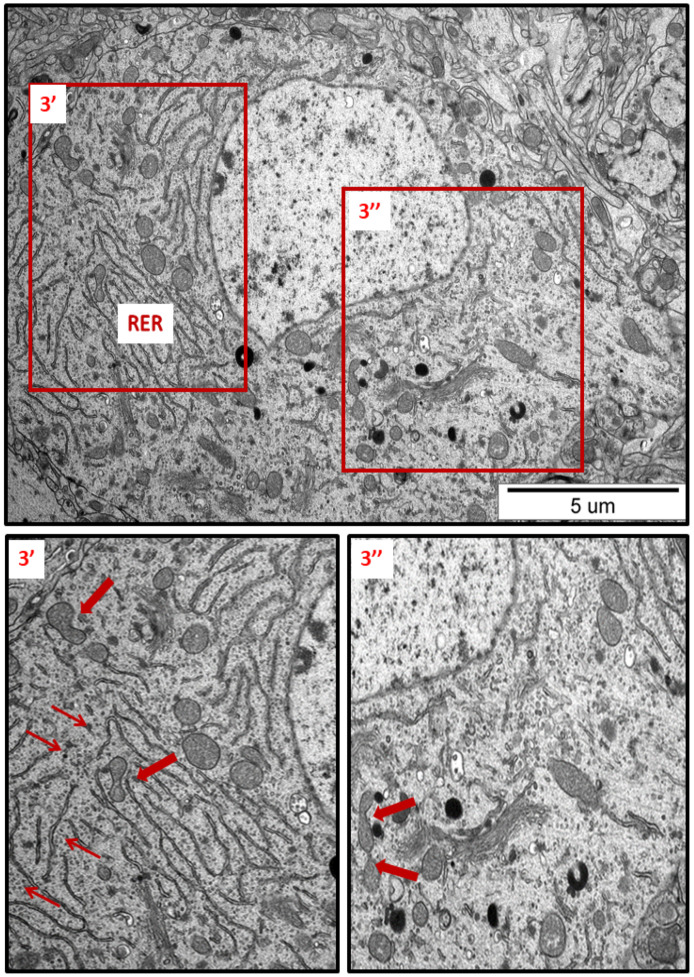
Representative TEM micrograph from hippocampal sections of AgNP-exposed animals. An overview of neuronal cytoplasm with enlarged and thickened rough endoplasmic reticulum (RER) cisternae; detached ribosomes (thin arrows); fragmentation of mitochondria (arrows); lysosomes (dark organelles in inset 3″). Insets (3′, 3″) were magnified 2–3×. Images are typical for each of the 3 examined animals per group.

**Figure 4:**
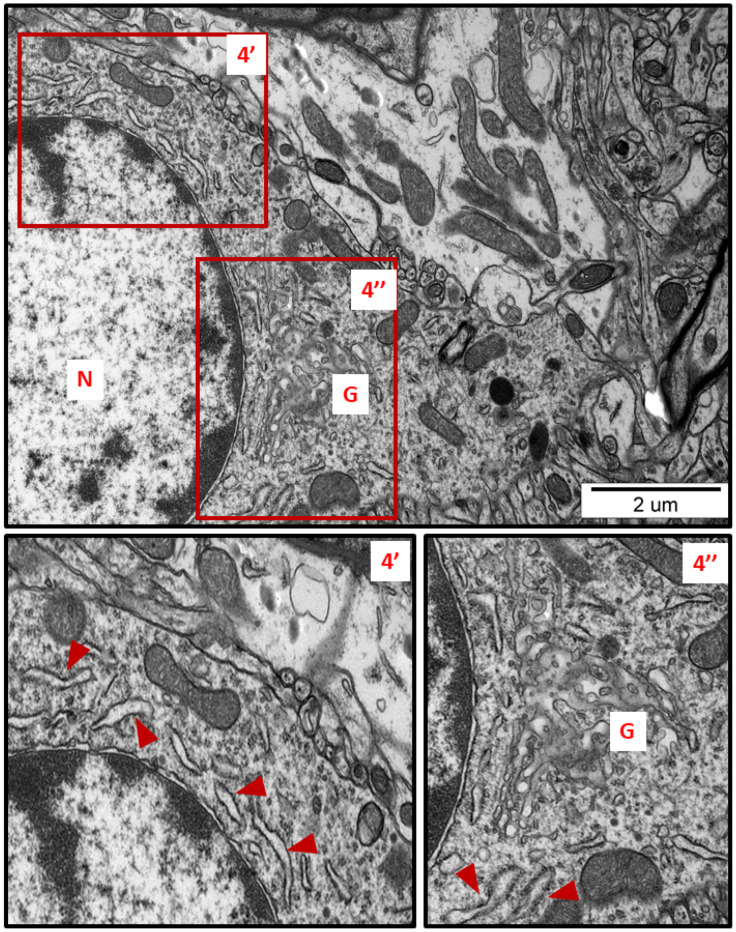
Representative TEM micrograph from brain tissue of AgNP-exposed animals showing swollen fragments of RER (arrowheads) and enlarged Golgi complex (G); N—nucleus. Insets (4′, 4″) were magnified 2×. Images typical for 3 animals per group.

**Figure 5:**
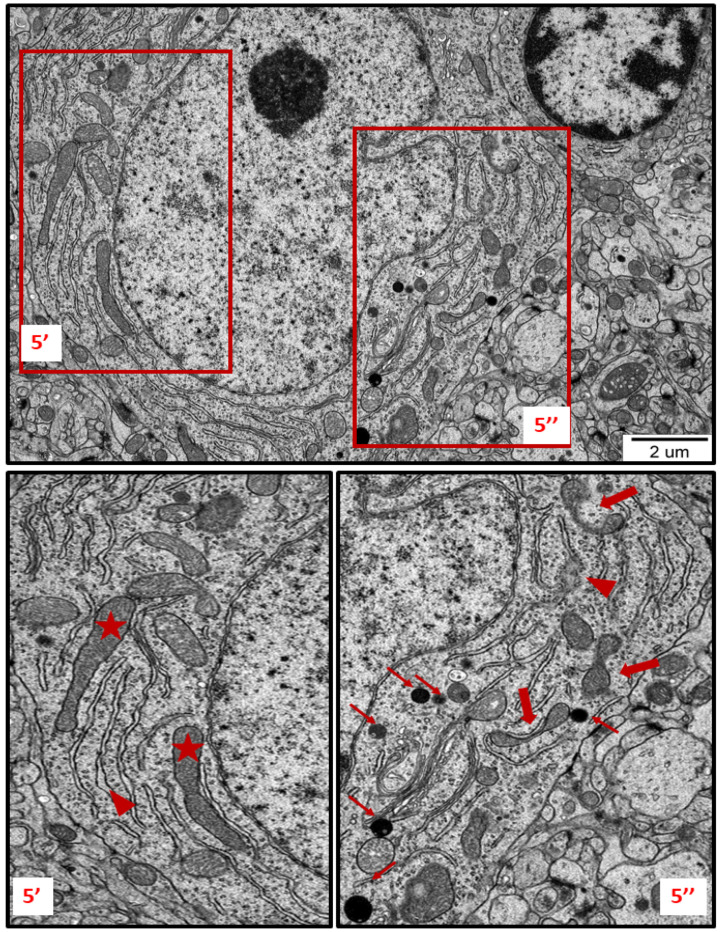
TEM micrograph showing ultrastructural changes in subcellular structures of neurons in brain sections obtained from AgNP-treated rats. Enlarged and swollen rough endoplasmic reticulum (RER) cisternae with blurred fragments (arrowheads) and detached ribosomes in the cytoplasm; elongated forms of mitochondria (asterisks); fragmentation of mitochondria (thick arrows); lysosomes (thin arrows). The insets (5′, 5″) are magnified 2–3×. Images are representative for each of the 3 animals.

**Figure 6:**
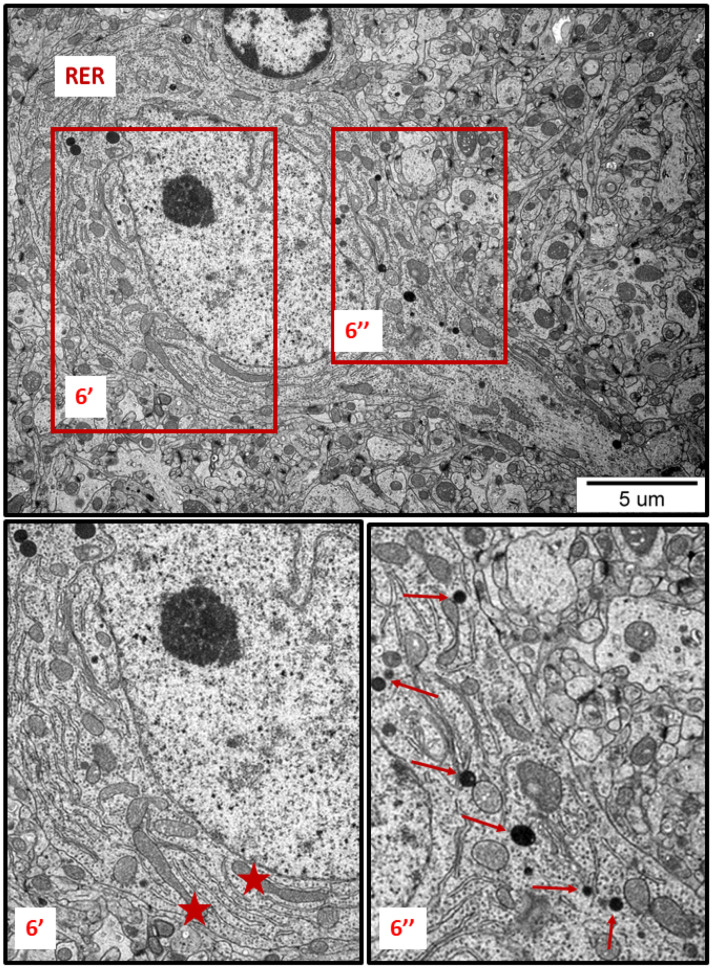
Representative TEM images from brain tissue of AgNP-exposed animals showing the ER stress in neuron. Thickened cisternae of rough endoplasmic reticulum (RER); elongated forms of mitochondria (asterisks); fragmentation of mitochondria (top of the inset 6″); lysosomes (thin arrows). The insets (6′, 6″) are magnified 2–3×. Images are representative for cerebral and hippocampal samples of each of the 3 animals.

In specimens obtained from control rat brains taken from cerebral cortex and hippocampus, the neuropil appeared normal, with properly organized cells and subcellular structures (Figure 1A,B). In brains of both AgNP- and Ag^+^-treated animals, pathological changes within neurons were identified early after the end of the 3-week exposure period (35 PND). Many neurons exhibited a proliferated ER. The “density” of the ER inside neurons was found to be enhanced, and the ER lumen was remarkably enlarged in many sections taken from both the cerebral cortex and the hippocampus (Figure 2, Figure 3, Figure 4, Figure 5 and Figure 6). Rough endoplasmic reticulum cisternae (RER) were found to be thickened and swollen (Figure 2, Figure 3, Figure 4 and Figure 5), and in some cases, short fragments of RER appeared as blurred structures (Figure 2 and Figure 5). In parallel, massive degranulation of RER membranes was observed, with detached ribosomes loosely accumulated in the cytoplasm of neurons (Figure 2, Figure 3 and Figure 5). Enlarged Golgi complexes were observed in some of the neurons (Figure 4).

TEM analysis also revealed variability in the shape of neuronal mitochondria. Dynamic changes in mitochondrial ultrastructure were found to be frequently present within a single cell. Some of the mitochondria were found to be fragmented (Figure 2, Figure 3, Figure 5 and Figure 6) whereas others appeared in an elongated form (Figure 5 and Figure 6), which is a known characteristic of ER-stress-induced autophagy. Many lysosomes were observed in the vicinity of fragmented mitochondria (Figure 3 and Figure 6).

**Figure 7:**
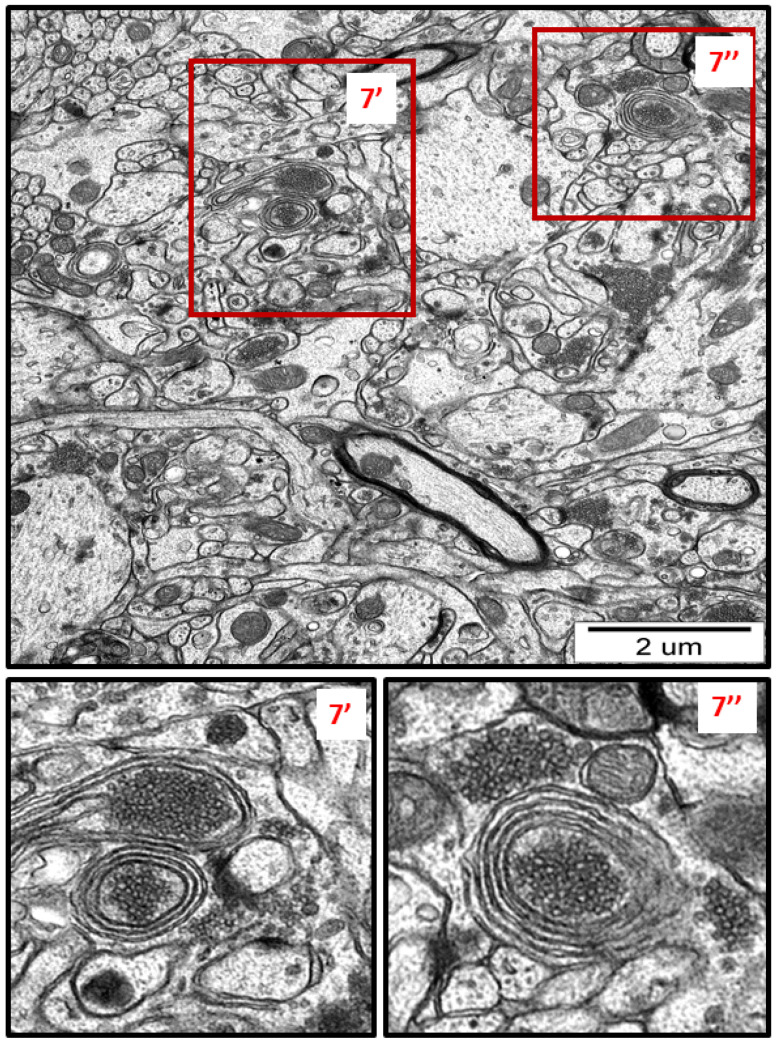
Representative TEM micrograph from AgNP-exposed rat showing the autophagic structures in hippocampus. Membranous whorls surrounding the remnants of cellular structures (7′, 7″). The insets (7′, 7″) are magnified 2–3×. Images are typical for each of the 3 examined animals per group.

**Figure 8:**
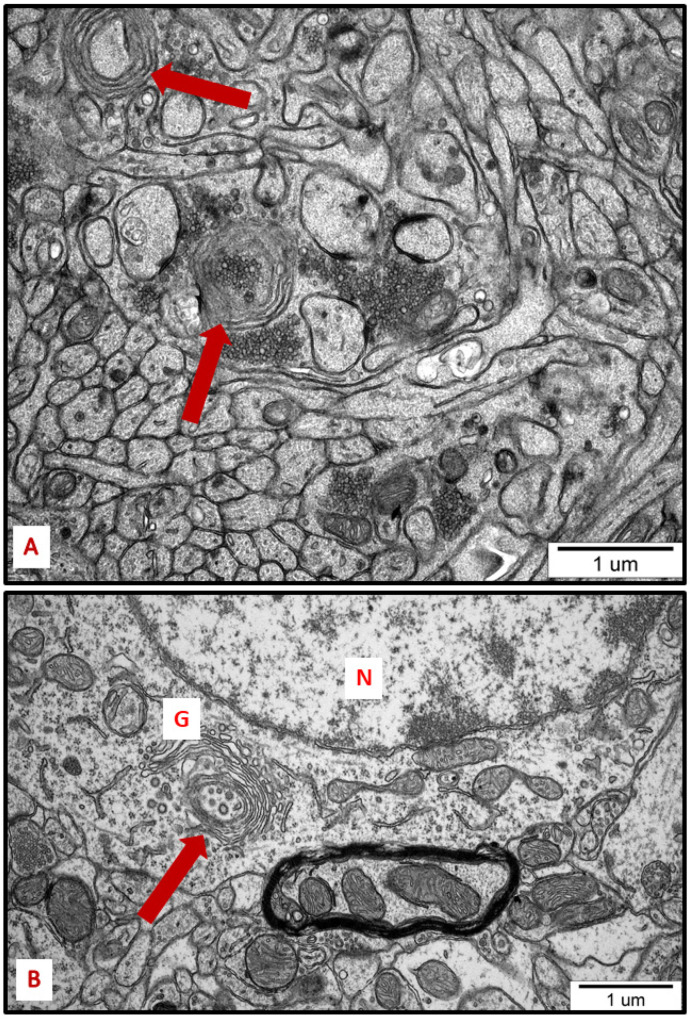
Representative TEM micrographs from hippocampus (**A**) and cerebral cortex (**B**) of AgNP-exposed animals showing the autophagic structures (arrows). Membranous whorls surrounding damaged subcellular structures are visible (**A**); autophagic structure formed from Golgi membranes (**B**). G—Golgi structure; N—nucleus.

Membranous whorls were also observed. These structures are also indicative of the autophagy process, and contain remnants of disturbed organelles that represent the formation of autophagosomes (Figure 7 and Figure 8). A similar pattern of autophagy structures has been previously reported by us in AgNP-exposed adult rat brain tissue in connection with synaptic degeneration [7,18]. The profile of ultrastructural changes in neuronal RER and mitochondria was found to be generally similar regardless of the silver form administered. However, elongated mitochondria and autophagy structures were more frequently observed in AgNP-exposed rats.

### 2.3. ER-Stress-Dependent Activation of the UPR upon AgNPs Exposure

**Figure 9:**
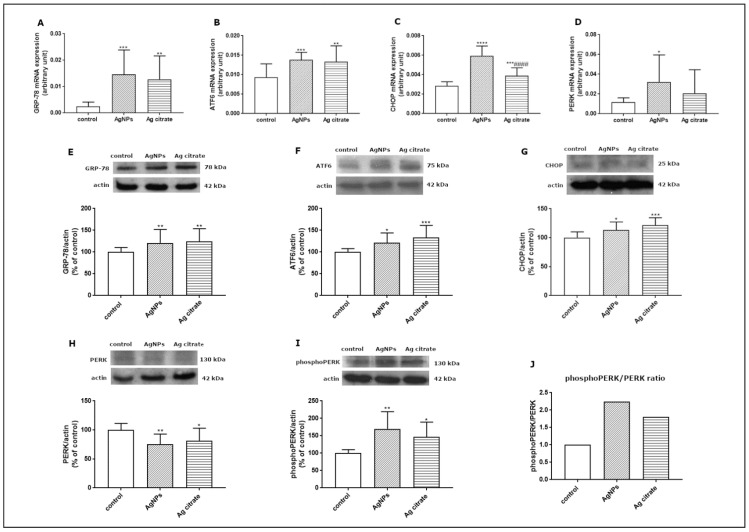
Expression of selected UPR-related proteins: GRP78 mRNA (**A**), ATF6 mRNA (**B**), CHOP mRNA (**C**), and PERK mRNA (**D**) in the brain of control, AgNP-, and Ag^+^-exposed rats. The mRNA levels were determined by qPCR and normalized against actin. The graph shows the results as mean ± SD from arbitrary units obtained from four independent experiments performed using 4 distinct rats per group. * *p* < 0.05, ** *p* < 0.01, *** *p* < 0.001, **** *p* < 0.0001 significantly different vs. control, #### *p* < 0.0001 significantly different vs. AgNPs (ANOVA with post hoc Tuckey’s test). Relative expression of GRP78 (**E**), ATF6 protein (**F**), CHOP protein (**G**), PERK protein (**H**), and phosphoPERK (**I**) protein in the brain of control, AgNP-, and Ag+-treated rats. The comparative ratio of phosphoPERK to PERK protein (**J**). Representative immunoblots and graphs illustrating the mean ± SD of densitometric measurements of five independent immunoblots performed using four distinct animals per group. The relative density was measured against β-actin as an internal standard and expressed as a percentage of control. * *p* < 0.05, ** *p* < 0.01, *** *p* < 0.001 significantly different vs. control (ANOVA with post hoc Tuckey’s test).

Since the TEM study revealed ultrastructural features indicative of the presence of ER stress in the brains of exposed animals, we further investigated this aspect at the molecular level. In order to verify whether AgNPs induce an unfolded protein response (UPR), which represents the predominant stress-responsive signaling pathway activated by ER stress, we studied the expression of several UPR-related proteins. We found that some of the UPR-regulated pathways are significantly activated at the transcriptional level. The expression of GRP-78 mRNA, the major chaperone protein that regulates the onset of the cellular response under stress conditions, was found to be significantly elevated in the brain tissue of both AgNP- and Ag^+^-treated rats compared to control (*p* < 0.001 and *p* < 0.01 vs. control) (Figure 9A). The level of ATF6 mRNA was also found to be increased in the AgNP-exposed group (*p* < 0.001 vs. control) and in the Ag^+^-exposed group (*p* < 0.01 vs. control) (Figure 9B). Similarly CHOP, a proapoptotic transcription factor which induces downstream PERK activation, was found to be significantly upregulated relative to both the control (*p* < 0.001) and Ag^+^-exposed group (*p* < 0.001) (Figure 9C) whereas PERK mRNA was increased exclusively in the AgNP-exposed group (*p* < 0.05 vs. control) (Figure 9D).

We also investigated the expression of the major UPR regulators at the translational level. Relative expression levels of GRP-78, ATF6, and CHOP proteins were found to be significantly elevated in both exposed groups compared to control (Figure 9E–G). In addition, we observed diminished expression of PERK protein (Figure 9H) with a concomitant increase in its phosphorylated form in both exposed groups (*p* < 0.01; *p* < 0.05 vs. control) (Figure 9I). The comparative phosphoPERK/PERK ratio was found to be above 2.0 and above 1.5 in animals exposed to AgNPs and Ag^+^, respectively, confirming increased phosphorylation of this regulatory protein and indicating UPR activation (Figure 9J).

## 3. Discussion

### 3.1. Ultrastructural and Molecular Determinants of ER Stress in Neurons under Conditions of AgNPs Exposure

The nervous system is particularly sensitive to toxic insults. Thus, even low-level exposure to toxic substances can lead to deleterious effects relative to other organs. Moreover, there is a tendency for AgNPs to accumulate in the nervous tissue over time [9].

When reaching the cell, nanoparticles interact with the components of the extracellular matrix and the plasma membrane and enter the cell via the mechanism of endocytosis. They are engulfed by membrane invaginations forming endocytic vesicles, which are then transported to various intracellular compartments, exerting negative effects. Depending on the cell type and molecules involved in the process, endocytosis can be classified into several types, such as phagocytosis, clathrin-mediated endocytosis, caveolin-mediated endocytosis, clathrin/caveolae-independent endocytosis, and macropinocytosis [19].

We have determined that prolonged exposure of immature rats to a low dose of AgNPs leads to induction of neuronal ER stress in brains of exposed animals. We show several ultrastructural characteristics of cellular ER stress, such as enhanced proliferation and pathological alterations in ER membranes, as well as remodeling of mitochondrial morphology and the induction of autophagy. Using TEM, we observed dilation of cisternae, degranulation of ribosomes, and disintegration of ER membranes. The proliferation of the ER and various modifications to its structure usually occur in mammalian cells under stressful conditions [20] and may indicate disturbed protein folding processes. TEM measurements have indicated that expansion of the RER surface occurs in response to the overload of misfolded proteins [21]. Furthermore, swollen ER cisternae with membrane rearrangement observed at the ultrastructural level accompanied by upregulation of certain UPR-related proteins have previously been reported as indicators of ER stress [22].

In addition to the ER, other organelles such as Golgi apparatus, mitochondria, and lysosomes appear to undergo autoregulation processes during cellular responses to the stress [23,24]. ER overload and secretion of large amounts of proteins contribute to the functional augmentation of the Golgi apparatus, i.e., to enhanced post-translational modifications of proteins and their vesicular transport. The capacity of the Golgi apparatus is regulated by the cell in accordance with the current requirements, in the form of a Golgi stress response [23]. The enlarged Golgi apparatus observed by us in AgNP-exposed animals (Figure 4) confirms that the cell responds to overload of the ER by expanding the size of the cellular secretory machinery. Moreover, the presence of numerous small mitochondria (Figure 2, Figure 3 and Figure 5) provides an indication of ER stress, which has been shown to promote proapoptotic signaling through mechanisms involving mitochondrial depolarization and fragmentation [25,26,27]. Fragmented dysfunctional mitochondria then undergo a specific autophagic process known as mitophagy [28]. Interestingly, prolonged exposure to AgNPs has been previously reported to disrupt mitochondrial morphology and function in brains of adult rats [7].

The increased number of lysosomes (Figure 3, Figure 5 and Figure 6) also reflects a response to the stressful conditions, in which cellular processes of autophagy are initiated, concomitantly with an increased capacity of lysosomes, in order to degrade and recycle damaged biomolecules [29]. UPR, induced during perturbations in cell homeostasis, potently enhances the lysosomal clearance of the ER portions to prevent excessive ER expansion. This process protects cells from apoptosis [30].

High chemical and biological reactivity of nanostructures, including AgNPs, poses a risk of interactions with biomolecules. Indeed, upon contact with biological systems, AgNPs undergo nano–bio interactions and develop protein coronas on their surfaces as a result of reactions with cellular proteins [31]. AgNPs can change the spatial structure of proteins. For example, the impact of AgNPs on ubiquitin conformation has been shown, where increased β-sheet content of this protein was created due to the formation of inter-protein hydrogen bonds through partially unfolded protein regions [32]. Association of AgNPs with albumins from bovine and human serum was reported to decrease the number of α-helices in protein coronas [33]. As suggested, these dynamic interactions can potentially lead to protein unfolding and subsequent activation of the cellular stress response [34].

The results of our study revealed that AgNPs are capable of inducing ER stress, although specific mechanisms of protein-AgNPs interactions inside neurons are difficult to identify. We also found that the ultrastructural characteristics of ER stress are accompanied by upregulation of molecular markers of the stress-induced cellular response after exposure to AgNPs/Ag^+^. The mRNA of GRP78, the main chaperone protein responsible for initiating the response, was found to be significantly increased. Moreover, transcriptional overexpression of other molecular components of the UPR pathways, such as PERK, ATF6, and CHOP, was also found to be significant.

The combined actions of the sensor proteins (PERK, ATF6) provide prosurvival or proapoptotic outputs (CHOP) [15]. Transcriptional targets within the regulatory repertoire of ATF6 include, but are not limited to, promoting degradation of misfolded proteins [35] and protective ER chaperones, including GRP78 [36]. GRP78 also upregulates the expression of caspase-3, caspase-9, CHOP, cytochrome c, and Bax/Bcl-2, thus aggravating ER-stress-induced apoptosis [37]. The transcriptional and translational overexpression of the studied molecules overlap, with the exception of PERK. However, the decrease in the level of PERK protein was found to be accompanied by an increase in the level of its phosphorylated form (Figure 9H–J). This indicates activation of this regulatory protein and the PERK-mediated arm of UPR, such as CHOP, which was found to be overexpressed (Figure 9C,G).

In addition, the upregulation of genes involved in protein processing and activation of major UPR regulators upon ER stress also leads to transcriptional overexpression of genes related to the induction of autophagy and cell survival or death. These molecular pathways are activated in stressed cells to restore ER homeostasis by increasing the protein folding capacity of the ER and reducing protein loading, as well as determining the cell fate in a manner which depends on the stress conditions. Stress-adaptive autophagy or, alternatively, apoptosis can be triggered by common upstream signals [38]. CHOP is a proapoptotic factor activated by the PERK branch of UPR, which plays an important role in promoting cell death following ER stress by multiple mechanisms, and its expression level has been found to increase upon accumulation of unfolded proteins in the mitochondrial matrix [39]. The upregulation of CHOP observed in AgNP-exposed animals indicates proapoptotic output of ER-stress-induced UPR. Nevertheless, at the ultrastructural level, we did not observe features of neuronal death, such as dark cells. It is possible that the concomitant protective outputs provided by activation of the main UPR regulators and their combined actions exceed the proapoptotic factors.

### 3.2. The UPR-Mediated Induction of the Adaptive Response in Neurons of AgNP-Treated Rats

It is known that in response to stress, cellular structures such as the ER and mitochondria undergo morphological changes by remodeling their shape, form and/or dimension, as well as their molecular compositions [40]. Some of the ultrastructural features observed by us, such as diverse mitochondrial morphology and the presence of autophagy structures, reflect the UPR-mediated adaptive response of neurons to the AgNP-induced stress.

In brains of AgNPs/Ag^+^-exposed rats, in parallel to the observation of small mitochondria that probably underwent fragmentation, we also identified the presence of elongated mitochondria (Figure 5 and Figure 6). Interestingly, both morphological forms often appeared simultaneously in one neuron (Figure 6). This could be an indication of how the cell deals with stress. The process of protective mitochondrial elongation that occurs via the mechanism of stress-induced mitochondrial hyperfusion (SIMH) is an expression of activated mitochondrial biogenesis [41] and provides evidence of a cellular defense against AgNP-induced ER stress.

ER-stress-induced remodeling of mitochondrial quality occurs through the PERK arm of UPR [26]. Mitochondria are protected by PERK-integrated signaling that activates SIMH as a pro-survival mechanism, regulating mitochondrial morphology and metabolic activity [16]. Activation of SIMH suppresses pathological mitochondrial fragmentation and promotes mitochondrial functions such as ATP production to protect cells against pathological insults [41,42,43]. Pharmacological or genetic inhibition of PERK-regulated SIMH results in increased mitochondrial fragmentation and impaired activity of mitochondrial respiratory chain during ER stress [16]. In support of these data is the observation of upregulation of PERK in AgNP/Ag^+^-exposed rats and activation of this protein, evidenced by the increased PPERK/PERK ratio. The upregulated PERK, by protecting mitochondria against fragmentation, increases cellular energetic capacity to facilitate recovery from ER stress. We therefore conclude that AgNP-induced ER stress is counteracted by PERK-mediated enhancement of mitochondrial biodynamic.

Another pro-survival mechanism triggered by the cell under stress is autophagy, which is mechanistically related to ER stress [44]. All three UPR branches can directly induce autophagy and autophagosome formation during ER stress via activation of multiple genes regulating the expression of several autophagy inhibitors [45,46].

Autophagy represents a lysosomal degradation pathway critical for cell survival in response to stress [47]. Upregulation of this process is believed to be protective, inducing the recycling of damaged proteins and organelles to generate energetic components that sustain cell homeostasis [48].

Autophagy, which is activated downstream UPR, can maintain energy homeostasis in individual organelles and in the entire cell by increasing metabolic activity. Misfolded proteins, damaged organelles, and cellular substances are degraded in autophagolysosomes to restore energy for the biosynthesis of new macromolecules.

The role of autophagy in the toxicity of nanomaterials, including AgNPs, has been previously mentioned [49,50]. As reported, AgNPs are capable of inducing this process in the brain of adult rats exposed to a low dose of AgNPs [7], but can also block autophagy flux by interfering with lysosomal functions [51].

Undoubtedly, under our experimental conditions, ER stress induced by AgNPs is partially counteracted by UPR-mediated processes such as enhanced biodynamic of mitochondria and autophagy. It is challenging to determine the effectiveness of this compensation. However, our TEM analysis indicates that neuronal death via the CHOP-mediated apoptotic pathway is unlikely to occur.

In conclusion, we provide ultrastructural and biochemical evidence that prolonged exposure to a low dose of both AgNPs and Ag^+^ leads to the induction of ER stress in neurons of immature rat brains, as indicated by ultrastructural and molecular markers. We also show that UPR-mediated protective mechanisms are simultaneously promoted as evidenced by ultrastructural characteristics, such as remodeling of mitochondrial morphology through hyperfusion and the presence of autophagic structures.

## 4. Materials and Methods

### 4.1. Particulate Silver

AgNPs (10 ± 4 nm) in a form of a colloidal solution of nanoparticles suspended in an aqueous citrate buffer in a concentration of 0.02 mg AgNPs/mL were purchased from Sigma-Aldrich Chemical Co. (St. Louis, MO, USA; CAS No. 730785). According to the manufacturer, citrate buffer provides long-term stability and homogeneity of the product by preventing agglomeration of nanoparticles, as indicated by the following parameters: refractive index n20/D = 1.333, fluorescence − λ_em_ = 388 nm and full width at half maximum value (FWHM) = 59 nm. Several batches of these AgNPs were additionally characterized in our electron microscopic study, where the degree of dispersion and size distribution of AgNPs was previously assessed [18].

### 4.2. Animals and Experimental Design

All experimental procedures using animals were carried out in strict accordance with the EU Directive for the Care and Use of Laboratory Animals (Directive 2010/63/EU) in compliance with the ARRIVE guidelines, and were approved by the Local Experimental Animal Care and Use Committee in Warsaw (488/2017).

Two-week-old Wistar rat pups (n = 42) from the Animal House of the Mossakowski Medical Research Institute, Polish Academy of Sciences (Warsaw, Poland), were used in the study. At postnatal day 14 (PND 14), pups were randomly allocated into 3 groups (n = 14): (i) an experimental group treated with AgNPs, (ii) a positive control group treated with Ag citrate, and (iii) a negative control group treated with saline. Appropriate solutions were administered once daily at a dose of 0.2 mg/kg body weight (b.w.)/day for 21 consecutive days. An oral gavage with a gastric probe for small animals was applied (AnimaLab, Poznań, Poland). We have previously described the procedure of animal exposure in detail in [9].

A low dose of AgNPs (0.2 mg AgNPs/kg b.w./day), which is relatively close to a base level of environmental contamination, was administered to the animals. The dose was calculated based on the theoretical value of AgNPs for water compartments according to the literature data [52].

At 35 PND, after administration of the final dose of AgNPs/Ag^+^, animals were either sacrificed by decapitation to obtain tissues for biochemical assays or were anesthetized and perfused for TEM analysis.

### 4.3. Analysis of Silver Concentrations in Brain Samples

Rats were sacrificed by decapitation. Brains from three animals per group (n = 3) were collected and sent to the certified Laboratory (“ZdroChem” Sp. Z o.o., Biological and Chemical Research Centre, University of Warsaw, Warsaw, Poland) for measurement of silver concentration using inductively coupled plasma mass spectrometry (ICP-MS; Elan 6100 DRC Sciex, Concord, ON, Canada).

### 4.4. Ultrastructural Analysis of Brains by TEM

The rats were anesthetized with nembutal (80 mg/kg b.w.) and perfused first with 0.9% NaCl in 0.01 M sodium–potassium phosphate buffer (pH 7.4), and then with 2% paraformaldehyde and 2.5% glutaraldehyde in 0.1 M cacodylate buffer (pH 7.4). Brain samples were subjected to a routine method of tissue processing for electron microscopic analysis. The samples were post-fixed in 1% OsO_4_ solution, dehydrated in an ethanol gradient, embedded in epoxy resin (Epon 812), and cut into ultra-thin sections stained with 9% uranyl acetate and lead nitrate. A transmission electron microscope (TEM) (JEM-1200EX, Jeol, Tokyo, Japan) equipped with a digital camera MORADA and iTEM 1233 software (Olympus Soft Imaging Solutions, GmbH, Münster, Germany) was used for the study.

### 4.5. Analysis of Gene Expression by qPCR

After decapitation, the brains were isolated under sterile conditions, placed in liquid nitrogen, and stored at −80 °C. Total RNA was isolated from the brain samples using TRI Reagent (Sigma-Aldrich, St. Louis, MO, USA) according to the method of Chomczynski and Sacchi [53]. The quality and concentration of the RNA were verified by DS-11Fx nano-spectrophotometer/fluorometer (De Novix, Wilmington, DC, USA). cDNA was synthesized from 2 µg of total RNA using a reverse transcription kit (Life Technologies, Forest City, CA, USA). The specific primers were obtained from Life Technologies (Life Technologies, Forest City, CA, USA): GRP78 assay ID Rn00592059_m1, PERK assay ID Rn00581002_m1, ATF6 assay ID Rn01490844, CHOP assay ID Rn00492098_g1, actin assayID Rn 00667869_m1. Quantitative real-time PCR (qPCR) analysis was conducted on a Roche LightCycler^®^ 96 system, using 5 µL of RT product and TaqMan PCR Master Mix, primers, and TaqMan probe in a total volume of 20 µL. The reaction parameters were as follows: initial denaturation at 95 °C for 10 min and 50 cycles of 95 °C for 15 s and 60 °C for 1 min. Each sample was tested in triplicate. The fluorescence signal from a specific transcript was normalized against that of the reference gene (actin), and the threshold cycle values (∆Ct) were quantified by the ∆∆CT method.

### 4.6. Western Blot Analysis

Animals were sacrificed by decapitation at PND 35. Removed brains were washed in a cold phosphate buffer (pH 7.4) and homogenized in RIPA lysis buffer (10 mM Tris-HCl pH 7.5 containing 150 mM NaCl, 1% Nonidet P40, 0.1% SDS, 1% Triton X-100, PMSF 0.1 mg/mL) in the presence of inhibitor cocktail (1 µg/mL leupeptin, 0.1 µg/mL pepstatin, and 1 µg/mL aprotinin). The lysates were centrifuged at 13,000× *g* for 10 min at 4 °C. The supernatant was collected and used to measure the relative protein concentration via the routine WB method. Samples containing 50–100 μg of protein/lane were separated by SDS–PAGE and then transferred onto nitrocellulose membrane (AmershamTM ProtranTM Supported 0.45 µm NC). After blocking in 5% nonfat milk, the membranes were incubated overnight at 4 °C with primary antibodies: polyclonal anti-Phospho-PERK, 1:500 (Invitrogen, cat. no. PA5-40294, Waltham, MA, USA), polyclonal anti-PERK, 1:500 (Invitrogen, cat. no. PA5-99447), polyclonal anti-GRP78, 1:1000 (Invitrogen, cat. no PA5-34941), polyclonal anti-CHOP, 1:500 (Invitrogen, cat. no: PA5104528), polyclonal anti-ATF6, 1:500 (Invitrogen, cat. no PA5116494) and monoclonal anti-actin 1:500, (MP Biomedicals, branch: Warsaw, Poland, cat. no 08691002). Then, the membranes were incubated with secondary antibody conjugated with horseradish peroxidase (Sigma-Aldrich: cat. no A-9169, cat. no A-2304). Signals were detected using the chemiluminescence ECL kit and visualized by exposure of membranes to an X-ray HyperfilmTM ECL (GE Healthcare Life Sciences, cat. no 70487, Freiburg, Germany). The films were scanned using ImageScanner III (GE Healthcare, LabScan 6.0, Freiburg, Germany) and quantified using the Image Quant TL v2005 program.

### 4.7. Statistical Analysis

The results of the study are expressed as mean ± SD. The number of animals is indicated in the respective figure legend. Differences between groups were compared using one-way analysis of variance (ANOVA), followed by Tukey’s multiple comparison post hoc test. *p* < 0.05 was considered significant. All analyses were performed using GraphPad Prism Software, version 6.0 (San Diego, CA, USA).

## Data Availability

Data are available from the authors.
